# An International Online Survey on Oral Hygiene Issues in Patients with Epidermolysis Bullosa

**DOI:** 10.3390/dj13090398

**Published:** 2025-08-30

**Authors:** Giovanna Garuti, Giacomo Setti, Chiara Lucia Guidetti, Gaela Barbieri, Ugo Consolo, Pierantonio Bellini

**Affiliations:** Dentistry and Oral Maxilla-Facial Surgery, Surgical, Medical, Dental and Morphological Sciences Department, University of Modena and Reggio Emilia, Via Del Pozzo 71, 41125 Modena, Italy; g.garuti@unimore.it (G.G.); giacomo.setti@unimore.it (G.S.); 284895@studenti.unimore.it (C.L.G.); 290632@studenti.unimore.it (G.B.); ugo.consolo@unimore.it (U.C.)

**Keywords:** epidermolysis bullosa, oral blistering, preventive dentistry, microstomia management, patient-reported outcomes

## Abstract

**Background**: Inherited epidermolysis bullosa (EB) includes a group of rare genetic disorders affecting the skin and mucous membranes. These disorders are characterized by extreme fragility and blister formation after minimal or no trauma. Oral and systemic manifestations vary by subtype; the more severe forms often present with extensive intra-oral blistering, scarring, microstomia, vestibular obliteration, ankyloglossia, and—in some cases—oral cancer. This study aims to collect data on oral-health practices and challenges in people with EB to inform preventive strategies and dental care. **Methods**: An international, structured online questionnaire with 31 items was distributed to individuals with a confirmed diagnosis of EB. The survey explored clinical and oral manifestations, home-care routines (oral hygiene and diet), experiences with dental professionals, and the impact of oral health on quality of life. **Results**: Eighty-two questionnaires were completed. Dystrophic EB was the most often reported subtype (69.5%). Most respondents (67.1%) experienced recurrent oral blisters and/or erosions. Many reported relying exclusively on soft foods and struggling with mechanical plaque removal because of microstomia and pseudo-syndactyly. Severe oral pain hindered effective brushing in 17% of participants. Hand contractures and microstomia interfered with oral hygiene in 74% and 31% of participants, respectively. Nearly 30% sought dental care only when in pain. Among those who did not attend regular check-ups or hygiene sessions (44.6%), the most cited reason was that dental clinics were inadequately equipped or trained to manage EB. **Conclusions**: Because dental procedures carry significant risks for patients with EB, preventive care should begin in early childhood. Yet many patients are still insufficiently informed about essential preventive measures and lack access to dental professionals trained in EB management.

## 1. Introduction

**Figure 1:**
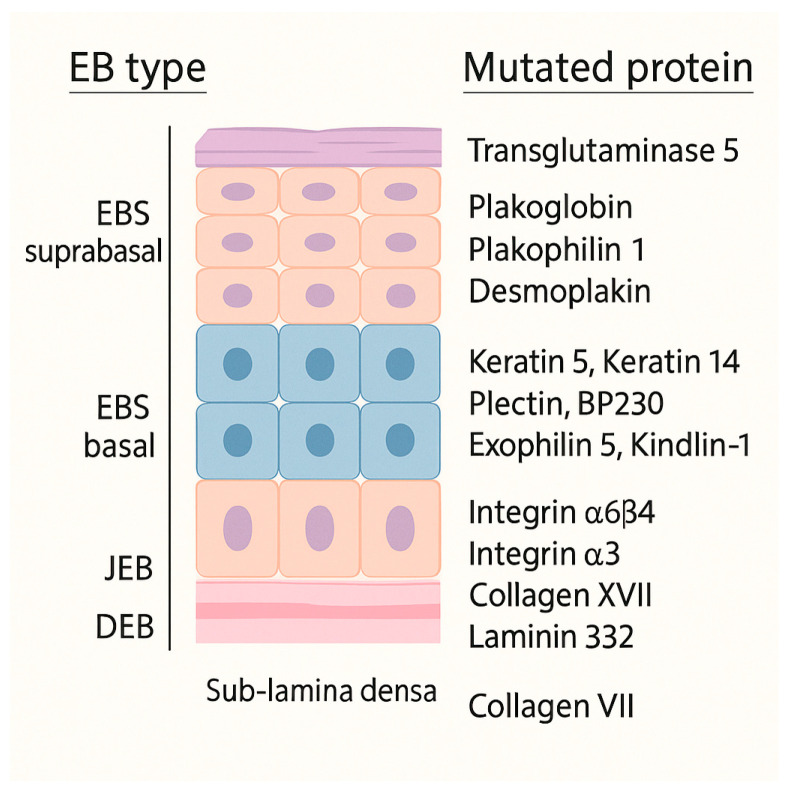
The illustration represents the epidermis with its basement-membrane zone, highlights the proteins involved in epidermolysis bullosa (EB), and marks the specific tissue plane where blisters arise in each EB subtype.

Epidermolysis bullosa (EB) encompasses a group of inherited genetic disorders caused by mutations in genes that encode structural proteins of the dermo-epidermal junction. These mutations weaken the anchoring of the epidermis to the dermis. Several consensus schemes have refined EB taxonomy over the past two decades. In 2014, Fine et al. issued updated diagnostic and classification recommendations that formalized ultrastructural and immunofluorescence criteria and served as the clinical standard for many years [1]. The 2020 classification system recognizes four major types (Figure 1), based on the level of skin cleavage, 35 subtypes, and five other skin-fragility disorders [2].

Tissue separation occurs in the basal keratinocytes in EB simplex (EBS), in the lamina lucida of the basal membrane in Junctional EB (JEB), into the dermis in Dystrophic EB (DEB) and in multiple levels in the Kindler EB (KEB). Moreover, classifying EB is challenging because the same gene (e.g., KRT5, KRT14, PLEC) can cause disease in either a dominant or a recessive fashion; this genetic overlap means that modern EB classification must consider both the level of skin cleavage and the specific gene involved [2,3].

Contemporary registry studies show that EB is still rare but more prevalent than early estimates, with point-prevalence ranging from 22 to 54 per million and incidence up to 67.8 per million live births in Europe and North America [4,5,6]. A 2024 Eastern-European registry reported 6.8 cases per million, underscoring persistent geographic variability [7].

**Figure 2:**
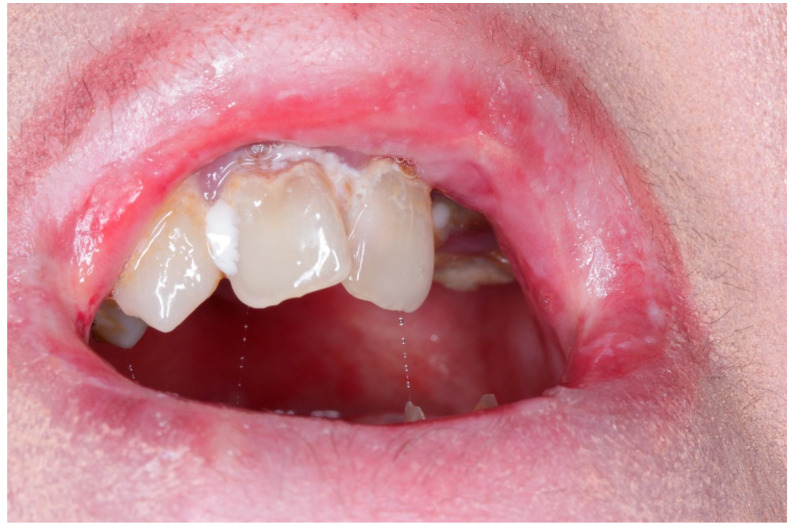
Intraoral view of a patient with EB, showing severe microstomia and mucosal blistering. Microstomia severely restricts access for routine oral hygiene, mucosal fragility and blistering further compromise effective plaque control due to the pain.

**Figure 3:**
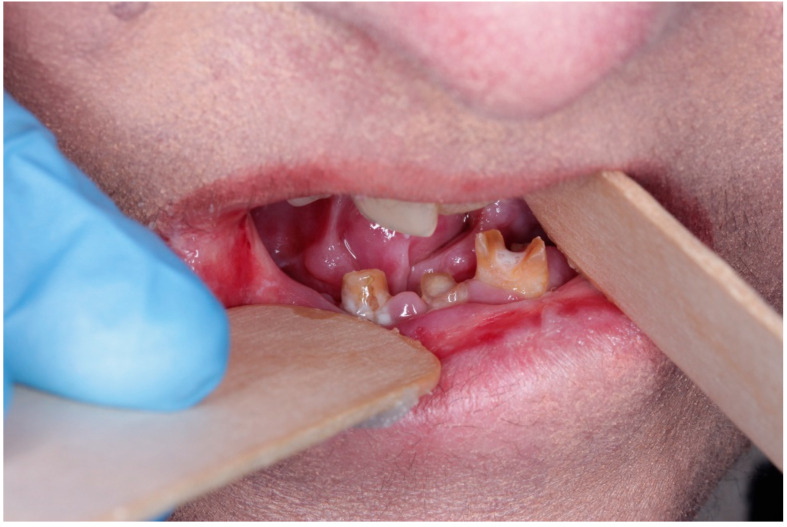
Extensive structural damage of the dentition in a patient with EB. The combination of limited mouth opening and pain from mucosal fragility leads to severe carious lesions and tooth loss, highlighting the need for early preventive intervention and tailored oral care strategies.

Despite heterogeneity across EB subtypes, recent multicenter and cohort studies confirm a consistently high burden of oral morbidity. Caries experience (dmft/DMFT) and plaque scores are significantly higher than age-matched controls [8,9,10]; microstomia, tongue contracture, and intra-oral blistering impair basic hygiene and nutritional intake (Figure 2 and Figure 3) [8,10]. Salivary flow may be reduced, further increasing cariogenic risk [10]. In EBS, and some milder JEB subtypes, recurrent blistering and mucosal ulceration dominate the clinical picture; severe JEB also presents generalized enamel hypoplasia that predisposes to early caries [8,9,10]. Dystrophic forms accumulate plaque and caries because microstomia, ankyloglossia and soft, carbohydrate-rich diets hamper hygiene [8,11], whereas Kindler EB is uniquely prone to early, severe periodontitis due to junctional-epithelial adhesive defects [12,13,14,15]. A 2024 prospective cohort found a robust correlation between objectively measured oral health (PhOX) and the EB-specific OHIP-14, with the greatest impact in dystrophic EB (rs = −0.54). These data confirm that oral sequelae translate into measurable reductions in oral-health-related quality of life (OHRQoL) [16]. The 2020 DEBRA clinical practice guideline provides evidence-based recommendations for prevention, anesthesia and rehabilitation [17], while a 2025 ERN-Skin position statement articulates referral pathways that integrate community dentists and specialist centers for complex care [18]. A 2024 scoping review nonetheless highlights persistent gaps in service delivery, especially for pediatric preventive visits and access to sedation/general anesthesia [19].

Most available data derive from chart reviews or tertiary-center cohorts; population-level information on daily oral-hygiene practices and patient-perceived barriers is scarce. To fill this gap, we conducted a cross-sectional, international, online survey of individuals with EB, aiming to describe self-reported oral-hygiene habits and difficulties, to quantify their perceived impact on daily life, and to generate patient-centered recommendations for clinicians.

## 2. Materials and Methods

To assess oral-hygiene challenges and their perceived impact in individuals with EB an international online survey was performed. Each participant completing the questionnaire once; no longitudinal follow-up or intervention was undertaken. No protocol registration was performed because this was a minimal-risk, fully anonymous survey.

A structured, 31-item, anonymous questionnaire available in Italian and English via Google Forms ^®^ (Google LLC, Mountain View, CA, USA) (https://docs.google.com/forms, Italian version: https://forms.gle/z3hQnaYoJkxu2Dwk8, English version: https://forms.gle/HtYhfJwL17ehRZGbA, all links accessed on 26 August 2025), was self-completed by participants.

Twenty five items included single- or multiple-choice questions, five used Likert-type scales (four- or five-point) to capture frequency or agreement, one item was open-ended to allow free-text comments on unmet needs. Frequency/impact items used a 4-point Likert format to avoid a neutral midpoint; agreement items used a 5-point scale to permit neutrality. Overall treatment satisfaction used a 1–10 numeric rating familiar to patients. The instrument was drafted by a multidisciplinary panel of dentists (3) and dental hygienists (2) experienced in EB management, and pilot-tested in 10 individuals but has not been formally validated.

The full questionnaire (Italian and English, with all response options) is available as Supplementary Material Files S1 and S2.

### 2.1. Ethical Considerations and Data Availability Statement

At the time of study design, ethical formal approval by the Ethics Committee (Area Vasta Nord, AVEN, Modena, Italy) was not needed for an anonymous questionnaire.

Participation was voluntary. Before enrollment, respondents must read an information page reporting the study purpose, data handling, anonymity, and their right to drop at any time; they then provided explicit electronic consent by selecting “I agree to participate.” E-mail addresses or other direct identifiers were not collected, IPs were not kept, and no combination of variables allows reasonable re-identification. So, the dataset is considered as anonymous information under the General Data Protection Regulation (EU 2016/679), Recital 26.

### 2.2. Survey Dissemination and Recruitment

The questionnaire was first drafted in Italian. Several Italian EB associations were contacted; however, despite initial interest, dissemination did not occur, and communication lapsed. DEBRA Südtirol–Alto Adige, an Italian regional EB patient association, agreed to disseminate the survey, and we proceeded with the Italian instrument. Although this region is bilingual (German/Italian), a German version was not produced within the study timeline owing to resource constraints. The association circulated the survey link via a dedicated WhatsApp group of registered members, the public Facebook page “DEBRA family”, and the association’s e-mail newsletter.

To increase geographic reach, the questionnaire was then translated into English and distributed globally. We contacted DEBRA branches asking them to post the anonymous link in compliance with their local privacy regulations. The following organizations/pages agreed to share the link publicly or in their newsletters: DEBRA International, DEBRA France, DEBRA Australia, EB Info World, Epidermolysis Bullosa Lounge, and DEBRA Austria’s quarterly newsletter. DEBRA USA and DEBRA UK declined participation owing to stricter privacy policies. The overall period in which participants received and completed the survey was between April and October 2023.

### 2.3. Sampling Considerations

Because EB is a rare condition and recruitment relied on convenience sampling through patient associations and social-media communities, no a priori power-based sample-size calculation was performed.

Recruitment through support networks and social media implies that participation depended on seeing the invitation and choosing to respond. This approach can introduce self-selection bias, for example, higher participation by individuals who are more engaged with EB communities, have stronger opinions, or greater symptom burden, while others (with milder disease, limited internet access, or different languages) may be under-represented. So, findings should be viewed as descriptive of respondents, not statistically representative of all people with EB.

Inclusion criteria were diagnosis of EB by a dermatologist or geneticist; age ≥ 5 years or active caregiver support for younger children; ability to complete the survey in Italian or English. Exclusion criteria included incomplete responses (defined as >20% missing answers).

### 2.4. Statistical Analysis

Response data were exported from Google Forms ^®^ to Microsoft Excel 365 (Microsoft Corp., Redmond, WA, USA). Frequencies, percentages, means and medians, as well as Pearson χ^2^ tests, were calculated directly in Excel.

## 3. Results

A total of 82 questionnaires met the inclusion criteria. Twenty-four respondents (29.3%) completed the Italian version and are therefore referred to as the Italian cohort (group 1); 58 respondents (70.7%) completed the English version and are grouped as the non-Italian cohort (group 2). The sample included 48% females and 52% males, with no sex difference in subtype distribution. The survey did not capture respondents’ country of residence or any similar geographic discriminant, so no finer breakdown beyond language version is available; the distinction therefore rests solely on whether participants completed the Italian or the English questionnaire.

EB-subtype distribution differed only slightly between language groups (Table 1): RDEB predominated in both (Italian 50%, non-Italian 57%). DDEB was less frequent among Italian respondents (8% vs. 16%), while JEB was more frequent (13% vs. 3%). A single non-Italian participant reported Kindler EB; acquired EB by four Italian participants. Across the entire sample, recessive dystrophic EB predominated (54.9%), followed by simplex EB (20.7%) and dominant dystrophic EB (14.6%). Junctional EB involved 6.1% of responses, Kindler EB 1.2%, and acquired/other forms 2.4% (Table 2). Thus, three in five respondents had a dystrophic form, and almost one in five had simplex EB.

Age distribution differed modestly between language groups (Table 2). The Italian cohort had proportionally more young children (0–5 years, 25%) and middle-aged adults (31–50 years, 29%), while the non-Italian cohort included more adolescents (6–15 years, 19%) and older adults (≥ 51 years, 19%). Young adults (16–30 years) accounted for one quarter of both groups (25% vs. 22%).

**Figure 4:**
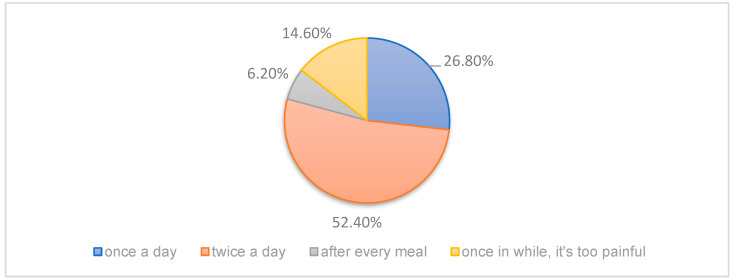
Reported frequency of adhesion to oral hygiene procedures (tooth-brushing).

In response to the question about oral hygiene practices, 43 participants (52.4%) reported brushing their teeth twice a day, 22 (26.8%) brushed once a day, and 14.6% informed that they brush only occasionally due to pain-related difficulties (Figure 4).

About quality of life and emotions within this sample, respondents who said oral problems affect them “all the time” (high impact) or “on many occasions” (moderate impact) selected anxiety and anger far more often than other. That can suggest a link between the perceived burden and negative effect, although the finding is exploratory. Oral-health problems have a clear, graded impact on daily life (Table 3). Almost half of respondents rated the burden as “all the time” (24.4%) or “on many occasions” (24.4%); 34.1% said it affected only a few activities and 17.1% reported no impact.

The emotional pattern mirrored this gradient. The most frequent feelings were anxiety about the future (32.5%) and anger (25%), followed by shame (16.4%), “None” (15.6%) and guilt (10.5%). Within the high-impact subgroup, anxiety and anger dominated (74% and 32%), while “None” was most common among those reporting little or no impact. A five-by-four Pearson χ^2^ test confirmed a significant association between perceived impact and emotions (χ^2^ = 37.1, d.f. = 12, *p* < 0.001). Although exploratory, because the emotion item allowed multiple selections, the data support a link between greater perceived burden and negative effect. With the limitation of multi-response bias, unknown direction as well as sample size, a suggestive pattern is noticeable.

**Figure 5:**
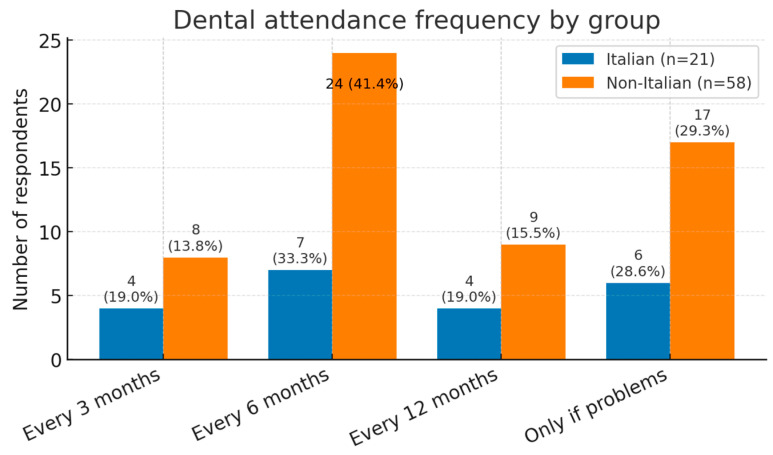
Recall-interval preferences: frequency of routine dental visits among survey respondents, split into subgroups. Italian responses for this item: *n* = 21/24; non-Italian: *n* = 58/58. Percentages are within-group among respondents.

In response to the question “How often do you visit the dentist for routine cleaning and check-ups?”, 31.7% of participants reported attending only when they experience dental problems. Regular attendance was reported to be 14.6% every 3 months, 38.5% every 6 months, and 15.9% every 12 months (Figure 5).

**Figure 6:**
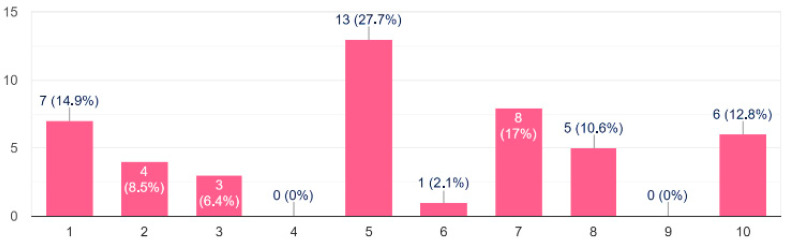
A total of 65 out of 82 participants answered about satisfaction with dental care, including fillings, root-canal therapy and prosthetic planning, rated on a 1-to-10 scale (1 = very negative, 10 = excellent). A score of 6–10 was given by 57% of Italian respondents and by 42.5% of non-Italian respondents, suggesting higher satisfaction among the Italian cohort.

Among patients who do not attend dental visits regularly, 40.6% identified the primary barrier as the lack of dentists adequately equipped or trained to manage subjects with epidermolysis bullosa. Other reported reasons included fear of pain (24.3%), excessive travel distance (15%), and earlier negative experiences with dental care (14.7%) (Table 4). Median overall dental-treatment rating was 5/10 (Interquartile Range, IQR, 3–7), showing moderate satisfaction and underscoring the need for EB-specific training. Overall satisfaction with dental treatment is resumed in Figure 6. Because this was an ad hoc 1–10 rating scale, with no formal validation, the figures should be interpreted as exploratory.

Table 5 shows that most patients in this study (67.1%) often experience intra-oral bullae, blisters, or erosions caused by EB. In 45.5% of these individuals, the lesions seriously hinder their ability to keep adequate oral hygiene at home, often making it extremely difficult or impossible.

Participants indicated the age at which they had their first dental examination: 42.7% attended before the age of three, 37.1% between three and six years, 19.5% after six years, and 6.1% had never visited a dentist.

**Figure 7:**
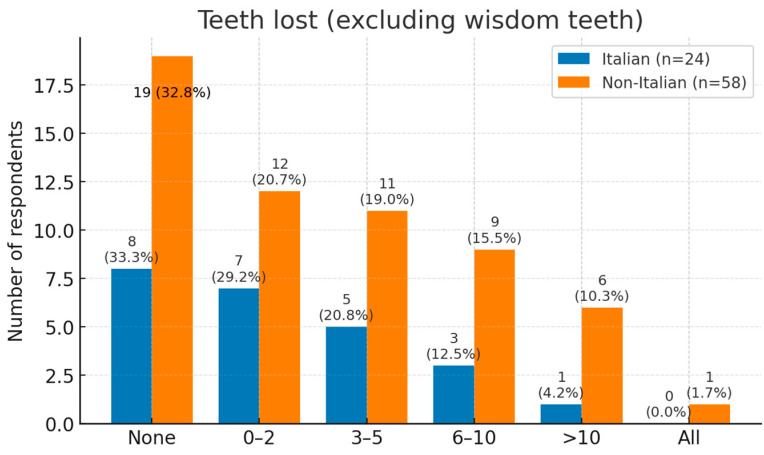
Reported response about tooth loss, highlighting a broadly similar distribution between language groups (respondents, *n* = 82). Percentages are within-group among respondents.

Tooth loss was commonly reported. One third of respondents had lost no teeth, while 23% had lost one or two. Moderate loss (3–10 teeth) affected 34%, and 8% had lost more than ten teeth; one non-Italian participant was edentulous (Figure 7).

Tooth crowding was reported by 45% of participants, yet only 16.2% of those had ever worn fixed braces (7.3% of the whole sample); all brace-wearers experienced mucosal blistering or pain (≥score 3/5), with four rating it 4–5 (significant pain/blistering), stressing the challenges of conventional orthodontics in EB.

Limited mouth opening (microstomia) was reported in 39% of patients in severe form and in 22% in mild form. Pseudo syndactyly—fusion or flexion contractures of the fingers—was reported by 23.2% of patients in severe form (*n* = 19) and 9.8% in mild form (*n* = 8) (Figure 4). Notably, 63% of those with microstomia also reported early tooth loss, supporting the view that restricted access for hygiene accelerates caries progression.

Both physical limitations hinder patients’ ability to perform effective oral hygiene at home. Patients with epidermolysis bullosa (EB) often show nutritional deficiencies leading to malabsorption, anemia, hypoalbuminemia, and impaired growth. Gastro-intestinal complications may involve any segment of the digestive tract; esophageal stenosis is the most serious because it restricts the texture and consistency of foods that can be swallowed. In the present study, only 37.8% of patients were under the care of a nutritionist for a personalized diet. Moreover, 64.2% reported that their nutritionist had never offered guidance on foods and beverages that protect, or may harm, oral and dental health. The foods least often consumed by Italian patients were raw vegetables, steak, and fruit (fresh or blended). Among non-Italian patients the least-consumed items were raw vegetables, crackers/crisp bread, steak, and sugary drinks.

With respect to texture-modified diets aimed at preventing oral blisters, only one-quarter of both Italian (25%) and non-Italian (25.9%) respondents could eat food of all consistencies without difficulty. One-third of Italian patients (33.3%) were limited to soft or liquid foods, compared with 10.3% of non-Italian patients. In the non-Italian group, 6.9% relied exclusively on artificial nutrition delivered via percutaneous endoscopic gastrostomy (PEG).

## 4. Discussion

Inherited epidermolysis bullosa (EB) exemplifies the interface between genodermatoses and oral health. Mutations in at least 20 genes—including COL7A1, KRT14, COL17A1 and FERMT1—impair epithelial adhesion at different ultrastructural levels, producing mucosal fragility that varies by subtype [2,20]. Common oral manifestations include microstomia, recurrent ulceration, scarring, ankyloglossia, periodontal disease, vestibular obliteration, and increased risk of oral squamous-cell carcinoma.

Hard tissues may present with enamel hypoplasia, caries, maxillary atrophy, delayed eruption, and malocclusion [17]. In subtypes with intrinsic enamel defects, tooth damage is difficult to avoid; moreover, limited mouth opening and mucosal blistering hinder effective oral hygiene and thereby increase caries risk [21].

Our survey of 82 individuals, currently the largest patient-reported dataset on everyday oral care in EB, documents and extends several genotype-phenotype observations that have been described since 2020.

As a cross-sectional, anonymous, convenience-sample survey in a rare disease, our data are liable to self-selection biases, language restriction, and the absence of a defined sampling frame. Diagnoses were self-reported and QoL instruments were not validated EB-specific tools in this survey. Togo et al. highlighted similar instrument heterogeneity and urged uptake of EB-specific tools such as QoLEB and InToDermQoL [22]. Unlike the cross-sectional clinical cohorts aggregated in the review, our survey recruited through patient-support networks, capturing perspectives that may be under-represented in hospital-based samples. In our view, these findings complement, rather than duplicate, the evidence synthesized in that review. Regarding language restrictions, the survey was available only in Italian and English. As a result, individuals who speak other languages—particularly from Latin America, Asia, and parts of Africa where EB prevalence is unknown, are under-represented. Language restriction may bias the sample toward higher educational level, greater digital engagement, and possibly easier access to care. Future studies should offer validated versions in additional languages or use registry-based sampling to obtain a more globally representative EB population.

With adequate preventive care, including a proper diet, professional cleaning, and regular examination, damage to both the primary and permanent dentitions can be mitigated [23]. In our study, 69.5% of patients were diagnosed with dystrophic EB (DEB), in which mutations in COL7A1 disrupt type VII collagen. Of these, 54.9% had recessive DEB (RDEB) and 14.6% had dominant DEB (DDEB). This aligns with the 2020 systematic review of 745 EB cases, which found that RDEB and JEB consistently scored lowest across generic and EB-specific QoL instruments [22]. DEB and junctional EB (JEB) are the subtypes most associated with oral complications; 67.1% (55 individuals) reported frequent intra-oral blistering. Pain caused by these lesions substantially interfered with daily oral hygiene: 36.4% of respondents said that it “greatly” affected their ability to clean their teeth, and 12.7% described the impact as “overwhelming”. Scarring and fibrosis of the perioral tissues lead to microstomia, which further complicates oral care. In our cohort, 39% of patients had severe microstomia and 22% had moderate limitations. This condition contributes significantly to caries development in both children and adults; consequently, all EB patients should be managed as individuals at high caries risk [24].

Tooth decay is particularly common in DEB. Poor oral hygiene and the high-carbohydrate diet needed to meet increased energy needs are the principal etiological factors. When painful blistering is present, patients often eat slowly and more often, prolonging exposure to cariogenic substrates. Impaired tongue mobility and vestibular obliteration reduce natural clearance and further exacerbate caries risk [25]. Our data shows that 54.9% of patients were not under the care of a nutritionist for personalized dietary management. Among those who were (37.8%), 64.2% reported that their nutritionist had never provided advice on food and drink choices that protect oral health. This reliance on calorie-dense, cariogenic foods mirrors the nutritional challenges summarized by Zidório et al., who emphasized early dietetic referral and high-protein supplementation, particularly for RDEB and JEB, to offset growth failure and anemia [26].

Although our survey did not directly measure periodontal parameters, the combination of compromised oral hygiene (due to microstomia, pain, and limited manual dexterity) and frequent consumption of soft, carbohydrate-rich foods may plausibly increase susceptibility to periodontal disease in EB.

Given the well-documented early-onset periodontitis in Kindler EB and the unexpectedly high rate of self-reported gingival bleeding in non-Kindler subtypes [12,15], future work should recruit larger, multilingual samples, use validated instruments for self-report periodontal screening (e.g., the CDC/AAP 8-item instrument and deriving nomogram [27,28], the Periodontal Disease Self Report (PDSR) [29]) to estimate risk without examination, and incorporate longitudinal follow-up. Definitive periodontal diagnosis will still require clinical examination and direct measures, undertaken only when safe and feasible in specialist settings.

Despite mucosal fragility, toothbrushing is possible for all patients when an ultra-soft, small-headed brush is used and moistened beforehand. During episodes of severe oral pain, alternative methods—such as cotton-tipped swabs, micro-brushes, or moistened gauze—can be used temporarily [30]. Significant functional limitation of the hands due to cutaneous scarring and joint contractures was reported by 23.2% (19/82) of patients, who experienced difficulties holding utensils or a toothbrush. Pseudo-syndactyly further impairs manual dexterity, needing caregiver help for daily oral hygiene: 20% of patients needed occasional help and more than 10% were completely dependent. Effective care for EB demands a multidisciplinary approach.

Patients should be referred to a pediatric dentist at once after diagnosis—ideally between three and six months of age, before oral lesions appear and before tooth eruption. Early counseling should cover diet, fluoride use, oral-hygiene instruction, and the oral manifestations of EB [17,18].

In our study, 42.7% of patients received their first dental examination before the age of three, 31.7% between three and six years, 19.5% after six years, and 6.1% had never visited a dentist. Recall intervals should be tailored to individual plaque levels and caries risk: while some patients need appointments every three to six months, others require monthly review [30]. Nevertheless, only 14.6% of our patients attended every three months, 39% every six months, and 15.9% once a year.

Among those who did not attend regularly, 44.6% cited inadequate preparedness of dental offices as the main barrier, and 24.3% feared pain. Almost half of the participants (39/82) reported being turned away by a dentist who felt unable to treat them. Comparable access problems have been documented outside dentistry; in a national U.S. survey, 19% of EB patients reported at least one emergency-department visit and one-quarter of caregivers said healthcare demands forced changes in work or education [31].

Structured, guideline-driven referral pathways offer a practical solution to these access gaps. The 2025 ERN-Skin position statement now provides six consensus-based dental-care pathways stratified by EB subtype and age, specifying first-referral age, recall frequency, and specialist roles for JEB, RDEB, Kindler and simplex forms [18].

Regarding psychosocial impact and emotional profile, our findings are in line with broader EB health-related QoL (HRQoL) literature, yet they highlight mouth-specific barriers that generic HRQoL tools often overlook. The survey confirms that oral sequelae of EB are not merely clinical inconveniences but carry an emotional signature that scales with the self-perceived impact on daily life. When the single-choice “impact on life quality” item was cross-tabulated with the multi-choice emotion list (Table 4), a 5 × 4 Pearson χ^2^ test showed a highly significant association (χ^2^ = 37.1, d.f. = 12, *p* < 0.001). Because the emotion item allowed multiple selections, this χ^2^ is exploratory.

**Figure 8:**
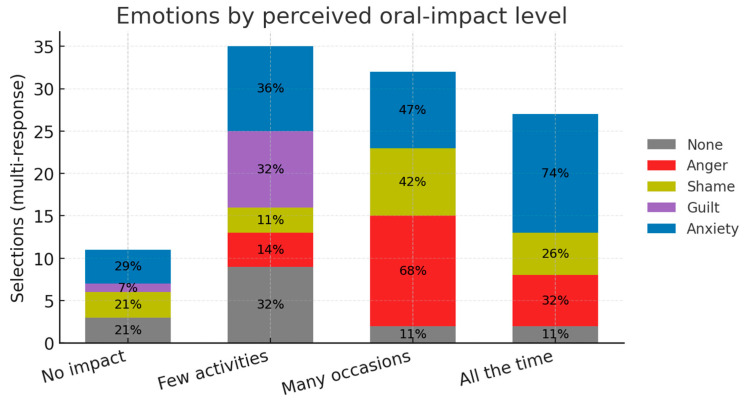
Emotions by perceived oral-impact level. Stacked bars show, within each impact category, the percentage of respondents who selected each emotion (multi-response: totals can exceed 100%). Emotions: none (gray), anger (red), shame (olive), guilt (purple), anxiety (blue). Respondents per category: no impact *n* = 14; few activities *n* = 28; many occasions *n* = 19; all the time *n* = 19. Labels inside segments are percentages. A χ^2^ test indicated an association between emotions and impact level (χ^2^ = 37.1, df = 12, *p* < 0.001); this is exploratory due to multiple responses.

The gradient is illustrated in Figure 8, where anxiety and anger dominate at the ‘high’ and ‘moderate’ impact levels, while the ‘None’ category is most frequent when impact is rated low or absent.

Three major patterns stand out: respondents who said oral problems affected them “all the time” or “on many occasions” selected anxiety (74% and 47%, respectively) and anger (32% and 68%) far more often than any other feeling. This echoes the qualitative work of El Hachem et al. [32], where anticipatory anxiety around mealtimes and frustration with repeated dental trauma were dominant themes.

Among those reporting “no impact at all, ”79% chose “None”, while anger was never chosen. A similar, though less pronounced, pattern was seen in the “few activities” group.

About oral-health-related quality of life (QoL), about 50% of patients were concerned about the aesthetics of their smile, while one-third prioritized function. Oral complications had a marked impact on QoL: 48.8% reported that dietary restrictions or missing social meals with friends affected them substantially, and only 17% reported no impact. A mixed-methods study of French children with EB likewise found markedly reduced COHIP-34 scores and interview themes of pain, dietary restriction and social exclusion, showing that OHRQoL impairment is clear from early childhood EB individuals with intact occlusion and minimal blistering report HRQoL scores comparable to population norms [33]. Pain-linked anxiety and depressive feelings are not unique to our cohort; the same pattern predominated in seven of the twelve studies considered by Togo et al. [22] These pediatric findings merge with the OHIP-14 data in adolescents reported by Joanning et al. [16] and with our present adult cohort, suggesting a life-course persistence of psychosocial burden.

Feelings of social embarrassment were reported across all impact bands, suggesting that even isolated aesthetic concerns, such as enamel opacities in JEB, can provoke psychosocial distress, consistent with recent photo-elicitation interviews in children with EB [21]. Since wound care and pain management generate anxiety and other negative emotions for both children and parents, psychological support is essential.

Because the emotion question allowed multiple selections, respondents could appear in more than one row of the contingency matrix, and the present χ^2^ should therefore be viewed as exploratory rather than confirmatory. Nevertheless, the gradient we saw aligns with general models of chronic illness, where perceived functional limitation is a stronger driver of negative affect than objective clinical severity. Future studies should confirm this relationship using single-emotion scales or DES/VAS scales, and longitudinal designs to evaluate whether successful preventive programs can shift both oral-impact ratings and emotional profiles over time.

## 5. Conclusions

This questionnaire-based study offers the largest patient-reported snapshot to date of oral complications in epidermolysis bullosa (EB). Across 82 respondents, dystrophic EB dominated (69.5%), and two-thirds experienced frequent intra-oral blistering that significantly hindered daily hygiene. Severe orofacial scarring and hand contractures further compounded caries risk. Early, prevention-centered dentistry appeared as the common denominator of good outcomes. From infancy, EB patients should enter a multidisciplinary pathway in which pediatric dentists deliver tailored hygiene instruction, fluoride therapy, pit-and-fissure sealants, and diet counseling that balances caloric need with cariogenic exposure. Where manual dexterity is limited, caregivers must be trained to help with ultra-soft brushes or moistened swabs. Maintaining a functional dentition is critical for adequate nutrition and quality of life; therefore, recall intervals must be individualized—monthly for high-plaque or high-caries patients, three- to six-monthly for others. Half of the cohort had been refused treatment by a dentist, underscoring the need for EB-specific training and clinic preparedness.

Based on our findings, and in keeping with the 2025 ERN-Skin oral-health pathways for EB, we recommend early referral of EB patients to dental professionals, since childhood; prompt evaluation and referral by dental professionals with documented experience in rare-disease management or to the nearest EB-designated center; EB-specific training for dental teams to prevent treatment refusal and ensure safe operative procedures; integration of dietary counseling, explicitly addressing cariogenic and periodontal risk into routine dental care plans; structured psychological support throughout childhood and adolescence to foster acceptance of their condition, reduce anxiety/anger and self-guilt, and facilitate adherence to oral-health routines.

Rigorous preventive protocols, early referral, and close collaboration between specialists can reduce the considerable oral disease burden in EB and improve patients’ daily functioning and psychosocial well-being.

## Figures and Tables

**Table 1:** Age distribution of study participants (Group 1 (G1): respondents from Italy; Group 2 (G2): respondents from All Other Countries).

Age Group	G1	% G1 (*n* = 24)	G2	% G2 (*n* = 58)
**0–5 yr**	6	25.0	7	12.1
**6–15 yr**	2	8.3	11	19.0
**16–30 yr**	6	25.0	13	22.4
**31–50 yr**	7	29.2	16	27.6
**≥51 yr**	3	12.5	11	19.0
**Total**	24	100	58	100

**Table 2:** Overall epidermolysis bullosa (EB) type distribution in study participants.

*EB Form*	*n*	% of Total
*Recessive dystrophic EB (RDEB)*	45	54.9
*Simplex EB (EBS)*	17	20.7
*Dominant dystrophic EB (DDEB)*	12	14.6
*Junctional EB (JEB)*	5	6.1
*Kindler EB (KEB)*	1	1.2
*Acquired/other EB*	2	2.4

**Table 3:** Responses to the questions about emotions related to oral-health problems and their impact on quality of life.

**Group**	**None** ***n* (%)**	**Anger** ***n* (%)**		**Shame** ***n* (%)**		**Guilt** ***n* (%)**	**Anxiety About the Future** ***n* (%)**
1	3 (16.6)	12 (54.5)		9 (40.9)		1 (4.5)	8 (36.4)
2	12 (21.1)	12 (21.1)		9 (15.8)		9 (15.8)	29 (50.9)
**Total**	15 (15.6)	24 (25)		18 (16.4)		10 (10.5)	37 (32.5)
**Group**	**No Impact at All** ***n* (%)**		**On a Few Activities** ***n* (%)**		**On Many Occasions** ***n* (%)**		**All the Time** ***n* (%)**
1	3 (13.6)		5 (22.7)		7 (31.8)		7 (31.8)
2	11 (19)		23 (39.7)		12 (20.7)		12 (20.7)
**Total**	14 (17.1)		28 (34.1)		19 (24.4)		19 (24.4)

Question: Which emotions do you experience when thinking about your mouth? (You can select multiple answers). Question: How do oral problems caused by epidermolysis bullosa impact your quality of life (e.g., not being able to eat what you want, having to give up lunch/dinner with friends, etc.)?

**Table 4:** Responses to the questions about frequency of dental controls and why clinical checks are not regular. Question: How often do you visit the dentist/hygienist for cleaning and checking your teeth?

**Group**	**Every 3 Months** ***n* (%)**		**Every 6 Months** ***n* (%)**		**Every 12 Months** ***n* (%)**		**Only If I Have Problems** ***n* (%)**	
1	4 (19)		7 (33)		4 (19)		6 (28.6)	
2	8 (13.8)		24 (41.4)		9 (15.5)		17 (29.3)	
**Total**	12 (14.6)		31 (38.5)		13 (15.9)		23 (31)	
**Group**	**Fears of Pain** ***n* (%)**	**Too Far to Go** ***n* (%)**		**Dental Offices Are Not Equipped** ***n* (%)**		**Teeth Are Not My Priority** ***n* (%)**		**Previous Negative Experience** ***n* (%)**
1	6 (50)	4 (33.3)		6 (50)		0 (0)		0 (0)
2	12 (40)	7 (23.3)		20 (66.7)		4 (13.3)		10 (33.3)
**Total**	18 (24.3)	11 (15)		26 (40.6)		4 (5.4)		10 (14.7)

Question: If you do NOT go regularly for professional hygiene and control, what is the reason? (You can select multiple answers).

**Table 5:** Responses to the questions about the development of intraoral bullae and the experience of pain during at-home oral hygiene procedures. Question: Do bubbles, blisters, or erosions caused by EB develop in your mouth?

**Group**	**No, I Have Never Had Them** ***n* (%)**	**Yes, They Appear Very Frequently** ***n* (%)**	**I Had Them Only in Childhood** ***n* (%)**	**Only Rarely** ***n* (%)**
1	3 (12.5)	14 (58.3)	0 (0)	7 (29.2)
2	3 (5.2)	42 (72.4)	2 (3.4)	11 (19.0)
**Total**	6 (7.3)	55 (67.1)	5 (6.1)	16 (19.5)
**Group**	**Little** ***n* (%)**	**Moderate** ***n* (%)**	**A Lot** ***n* (%)**	**Overwhelming** ***n* (%)**
1	6 (27.3)	8 (36.4)	6 (27.3)	2 (9.1)
2	20 (36.4)	8 (14.5)	20 (36.4)	7 (12.7)
**Total**	26 (33.8)	16 (20.8)	26 (33.8)	9 (11.7)

Question: When you have intra-oral blisters, how much does pain interfere with your ability to maintain proper oral hygiene at home?

## Supplementary Materials

The following supporting information can be downloaded at: https://www.mdpi.com/article/10.3390/dj13090398/s1, File S1: complete questionnaire, Italian Language. File S2: complete questionnaire, English Language.
