# Supplementary material for: An International Online Survey on Oral Hygiene Issues in Patients with Epidermolysis Bullosa
Source: Dent J (Basel). 2025 Aug 30;13(9):398. doi: 10.3390/dj13090398 (PMC12468093; doi:10.3390/dj13090398)
Supplement: Supplementary file 1 [file dentistry-13-00398-s001.zip › Supplement_S1_Questionnaire_Italian.pdf]

Supplementary material:

S1, complete questionnaire, Italian Language

Full questionnaire used in the study. All questions list the exact response options. Frequency/impact items use a 4-point Likert scale without a neutral midpoint; agreement items use 5 points; one item is open-ended; treatment satisfaction uses a 1–10 numeric rating. Some questions allow multiple responses (indicated).

1. Q1. Che tipo di EB presenti?

Type: Scelta singola

- EB semplice (EBS)
- EB giunzionale (JEB)
- EB distrofica dominante (DDEB)
- EB distrofica recessiva (RDEB)
- EB di Kindler (KEB)
- EB acquisita

2. Q2. Qual è la tua fascia d’età?

Type: Scelta singola

- 0–5 anni
- 6–15 anni
- 16–30 anni
- 31–50 anni
- >50 anni

3. Q3. Quante volte al giorno pulisci i denti?

Type: Scelta singola

- 1
- 2
- dopo ogni pasto
- solo ogni tanto: è troppo doloroso

4. Q4. Chi si occupa della tua igiene orale a casa?

Type: Scelta singola

- io
- io ma con l’aiuto di qualcuno
- solo un’altra persona (genitore/caregiver)

5. Q5. Nella tua bocca si sviluppano bolle/vesciche e/o erosioni dovute all’EB?

Type: Scelta singola

- No, mai avute
- Sì, compaiono molto frequentemente
- Le ho avute da bambino ma poi non si sono più presentate
- Solo raramente

6. Q6. Quando sono presenti bolle/vesciche in bocca, quanto il dolore ti impedisce di svolgere una corretta igiene orale domiciliare?

Type: Scala Likert 4 punti (frequenza/impatti)

- poco
- abbastanza
- molto
- moltissimo

7. Q7. Hai problemi di limitata apertura della bocca (microstomia)?

Type: Scelta singola

- sì
- no
- solo un po’/parzialmente

8. Q8. Se presenti una apertura limitata, questo problema ti impedisce di svolgere una corretta igiene orale domiciliare?

Type: Scelta singola

- no
- posso farla ma non accuratamente
- sì

9. Q9. Hai problemi alle mani (chiusura e/o flessione delle dita) che ti impediscono di afferrare correttamente lo spazzolino?

Type: Scelta singola

- sì
- no
- a volte

10. Q10. A che età hai effettuato la prima visita odontoiatrica (dal dentista)?

Type: Scelta singola

- prima dei 3 anni
- tra i 3 e i 6 anni
- dopo i 6 anni
- mai

11. Q11. Se ti sei sottoposto a cure odontoiatriche (es. otturazioni, devitalizzazioni, protesi), su una scala da 1 a 10, come valuti nel complesso le esperienze che hai avuto?

Type: Scala numerica 1–10

- 1
- 2
- 3
- 4
- 5
- 6
- 7
- 8
- 9
- 10

Note: 1 = pessima, 10 = eccellente

12. Q12. Quando ti sottoponi all'ablazione del tartaro (pulizia dei denti), chi la esegue?

Type: Scelta singola

- il dentista

- l’igienista dentale
- dipende, a volte l’uno a volte l’altro
- non l’ho mai fatta

13. Q13. Per effettuare le terapie odontoiatriche ti sei rivolto a strutture ospedaliere o private?

Type: Scelta singola

- Ospedale
- Privato
- Entrambe

14. Q14. Ogni quanti mesi ti rechi dal dentista/igienista per la pulizia e il controllo dei denti?

Type: Scelta singola

- ogni 3 mesi
- ogni 6 mesi
- ogni 12 mesi
- solo se ho dei problemi

15. Q15. Se NON ti rechi regolarmente per i controlli e l’igiene professionale, qual è il motivo? (Selezione multipla possibile)

Type: Scelta multipla

- paura del dolore
- troppa strada / mancanza di risorse
- studi non attrezzati e/o non preparati per pazienti con EB
- la salute della bocca e dei denti non è una priorità
- precedenti esperienze negative

Note: Risposta multipla

16. Q16. Ti è mai capitato di non essere preso in cura perché il dentista non si riteneva in grado di gestire il tuo caso?

Type: Scelta singola

- sì
- no

17. Q17. Ti è stata spiegata l'importanza della prevenzione (sigillature, vernici al fluoro, cibi cariogeni)?

Type: Scelta singola

- sì, in modo approfondito
- in modo superficiale
- no, mai

18. Q18. Tra strumenti e prodotti per l'igiene orale in commercio, hai trovato quelli più adatti al tuo caso?

Type: Scelta singola

- sì, quelli che uso vanno bene
- no, non riesco a trovare quelli giusti per me
- uso quello che trovo, ma so di non pulire quanto dovrei

19. Q19. La tua dieta è varia o riesci a mangiare solo cibi morbidi per evitare la formazione di bolle nella mucosa della bocca?

Type: Scelta singola

- mangio tutti i cibi senza problemi
- mangio quasi tutto, evito solo i cibi troppo duri
- mangio solo cibi morbidi e liquidi
- mi nutro solo con nutrizione artificiale (PEG)

20. Q20. Tra gli alimenti elencati sotto, quali mangi con frequenza maggiore e quali raramente?

Type: Scale di frequenza per ciascun alimento (4 punti)

- Mai / Qualche volta / Spesso / Molto spesso per: cracker/chips/pane croccante; pasta/pane morbido/panini; puree di frutta; frutta fresca; latte/yogurt; insalata verde; verdure cotte; bistecca; carne macinata/polpette; dolci/snack; bevande zuccherate

Note: Valutare per ciascun alimento

21. Q21. Sei seguito da un nutrizionista per una terapia nutrizionale personalizzata?

Type: Scelta singola

- Sì
- No, non mi segue nessuno
- Cerco da solo informazioni su Internet

22. Q22. Se hai un nutrizionista che ti segue, ti ha spiegato quali cibi e bevande proteggono denti e mucose e quali sono dannosi?

Type: Scelta singola

- Sì
- No

23. Q23. Sui tuoi denti è presente fin dall'eruzione una parziale o totale assenza di smalto? (opacità, solchi, linee, macchie)

Type: Scelta singola

- Sì, evidente
- Sì, lieve
- No, lo smalto è normale

24. Q24. Hai sensibilità ai denti quando mangi o bevi cose particolarmente calde o fredde?

Type: Scelta singola

- Sì, spesso
- No, mai
- A volte, ma nella norma

25. Q25. I tuoi denti sono ben allineati o sono affollati?

Type: Scelta singola

- sono abbastanza allineati
- sono affollati

26. Q26. Se i tuoi denti sono affollati, hai mai messo l'apparecchio fisso (con le piastrine) per allinearli?

Type: Scelta singola

- sì, l’ho portato
- no, mai

27. Q27. Se hai portato l’apparecchio fisso, il contatto delle piastrine con le mucose ti ha causato bolle/vesciche e dolore?

Type: Scala 1–5

- 1 (nessun dolore)
- 2
- 3
- 4
- 5 (dolore intenso/vesciche)

28. Q28. Quanta importanza dai all’estetica del tuo sorriso?

Type: Scelta singola

- Ci tengo molto
- Mi importa di più l’aspetto funzionale
- Non mi interessa

29. Q29. Quanti denti hai perso per carie, infezioni o motivi parodontali? (Esclusi i denti del giudizio)

Type: Scelta singola

- nessuno
- da 0 a 2
- da 3 a 5
- da 6 a 10
- più di 10
- tutti

30. Q30. Quanto i problemi orali dovuti all’EB impattano sulla tua qualità di vita?

Type: Scala Likert 4 punti (impatto)

- per niente
- su poche attività
- in molte occasioni
- sempre

31. Q31. Quali emozioni provi quando pensi alla tua bocca? (risposte multiple)

Type: Scelta multipla

- ansia per il futuro
- rabbia
- vergogna
- senso di colpa
- nessuna

Note: Risposta multipla; analisi esplorativa

Abbreviazioni / Abbreviations

EB = epidermolisi bollosa / epidermolysis bullosa; EBS = EB semplice / simplex; JEB = EB giunzionale / junctional; DDEB = EB distrofica dominante; RDEB = EB distrofica recessiva; KEB = EB di Kindler; PEG = gastrostomia endoscopica percutanea; QoL = qualità della vita; OHRQoL = qualità della vita relativa alla salute orale.
