# Supplementary material for: An International Online Survey on Oral Hygiene Issues in Patients with Epidermolysis Bullosa
Source: Dent J (Basel). 2025 Aug 30;13(9):398. doi: 10.3390/dj13090398 (PMC12468093; doi:10.3390/dj13090398)
Supplement: Supplementary file 1 [file dentistry-13-00398-s001.zip › Supplement_S2_Questionnaire_English.pdf]

Supplementary material:

S2, complete questionnaire, English Language

Full questionnaire used in the study. All questions list the exact response options. Frequency/impact items use a 4-point Likert scale without a neutral midpoint; agreement items use 5 points; one item is open-ended; treatment satisfaction uses a 1–10 numeric rating. Some questions allow multiple responses (indicated).

1. Q1. What type of EB do you have?

Type: Single choice

- Simplex EB (EBS)
- Junctional EB (JEB)
- Dystrophic dominant EB (DDEB)
- Dystrophic recessive EB (RDEB)
- Kindler EB (KEB)
- Acquired EB

2. Q2. What is your age range?

Type: Single choice

- 0–5 years
- 6–15 years
- 16–30 years
- 31–50 years
- Over 50 years

3. Q3. How many times a day do you clean your teeth?

Type: Single choice

- 1
- 2
- after every meal
- just once in a while, it is too painful

4. Q4. Who takes care of your oral hygiene at home?

Type: Single choice

- me
- me but with someone’s help
- only one other person (parent/caregiver)

5. Q5. Do bubbles, blisters and/or erosions due to EB develop in your mouth?

Type: Single choice

- No, I never had them in my mouth
- Yes, they appear very frequently
- I used to have them in my childhood
- only rarely

6. Q6. When blisters are present in your mouth, how much does pain prevent you from performing proper oral hygiene at home?

Type: 4-point Likert (impact)

- little
- enough
- a lot
- overwhelming

7. Q7. Do you have problems with limited mouth opening (microstomia)?

Type: Single choice

- yes
- no
- partially / just a little

8. Q8. If you have limited mouth opening, is this preventing you from performing proper oral hygiene at home?

Type: Single choice

- no
- I can do it, but not accurately
- yes

9. Q9. Do you have problems with your hands (closing and/or flexing fingers) that prevent you from properly grabbing your toothbrush?

Type: Single choice

- yes
- no
- sometimes

10. Q10. How old were you when you had your first dental examination (at the dentist)?

Type: Single choice

- before the age of 3
- between 3 and 6 years
- after 6 years
- never

11. Q11. If you have undergone dental treatments (e.g., fillings, root canal treatment, dentures), on a scale of 1 to 10, how do you rate your overall experience?

Type: Numeric rating 1–10

- 1
- 2
- 3
- 4
- 5
- 6
- 7
- 8
- 9
- 10

Note: 1 = very bad, 10 = excellent

12. Q12. When you go to the dentist for professional oral hygiene (tartar removal), who performs the treatment?

Type: Single choice

- the dentist
- the dental hygienist
- it depends, sometimes one, sometimes the other
- I’ve never done professional oral hygiene

13. Q13. To perform dental therapies did you go to a hospital or to private clinics?

Type: Single choice

- hospital
- private clinic
- both

14. Q14. Every how many months do you go to the dentist/hygienist for cleaning and checking your teeth?

Type: Single choice

- every 3 months
- every 6 months
- every 12 months
- only if I have problems

15. Q15. If you do NOT go regularly for professional hygiene and controls, what is the reason? (multiple answers allowed)

Type: Multiple choice

- fear of pain
- too far to go / lack of resources
- dental offices are not equipped and/or prepared for treatment of EB patients
- teeth are not my first priority
- previous negative experiences

Note: Multiple response

16. Q16. Have you ever visited a dental office for professional hygiene and/or dental treatment and you did not get treated because the dentist thought they could not handle your case?

Type: Single choice

- yes
- no

17. Q17. Has a parent or a dentist/dental hygienist ever explained to you the importance of prevention (seals, fluoride varnishes, cariogenic foods, etc.)?

Type: Single choice

- yes, they spoke in depth
- just in a superficial way
- no, never

18. Q18. Among the tools and products for oral hygiene on the market, did you find the ones most suitable for you?

Type: Single choice

- yes, the tools I use are great
- no, I can’t find the right ones for me
- I use what I find, but I’m aware not to clean my mouth as well as I should

19. Q19. Nutrition plays an important role. Do you usually eat a variety of food or only soft foods to prevent blisters in the mouth’s mucosa?

Type: Single choice

- I eat all the food without problems
- I eat almost everything, I avoid only too hard foods
- I only eat soft, liquid foods
- I only feed with artificial nutrition (PEG)

20. Q20. Among the foods listed below, which ones do you eat frequently and which ones rarely?

Type: Frequency scales for each food (4 points)

- Never / Sometimes / Often / Very often for: crackers/chips/hard bread crusts; pasta/soft bread/sandwiches; fruit purees; fresh fruit; milk/yogurt; green salad; cooked vegetables; steak; minced meat/meatballs; sweets and snacks; sugary drinks

Note: Rate each food

21. Q21. Are you being followed by a nutritionist for a personalised nutritional therapy?

Type: Single choice

- Yes
- No, nobody follows me
- I search information on the internet by myself

22. Q22. If you have a nutritionist who follows you, has he/she ever explained what foods and drinks protect teeth and mucosa and which harmful ones should be limited?

Type: Single choice

- Yes
- No

23. Q23. Have you noticed, or been told, that there is a partial or total absence of enamel since the eruption of baby teeth? (opaque teeth, with cracks, depressions, lines and/or spots)

Type: Single choice

- Yes, obvious
- Yes, mild
- No, enamel is normal

24. Q24. Do you have tooth sensitivity (pain for a few seconds) when you eat or drink things that are particularly hot or cold?

Type: Single choice

- Yes, often
- No, never
- Sometimes, but not too worrying

25. Q25. Are your teeth aligned or are they crowded?

Type: Single choice

- they are fairly aligned
- they are crowded

26. Q26. If your teeth are crowded, have you ever worn fixed braces (with brackets) to align them?

Type: Single choice

- Yeah, I got braces
- No, never

27. Q27. If you have a fixed brace, does the contact of the bracket with the mucous membranes cause blisters and pain?

Type: Scale 1–5

- 1 (no pain)
- 2
- 3
- 4
- 5 (severe blistering/pain)

28. Q28. How much importance do you give to the aesthetics of your smile?

Type: Single choice

- I care a lot about the look of my teeth
- I care more about the functional aspect rather than the aesthetic
- I don’t care, I have more important issues to take care of

29. Q29. How many teeth did you lose due to tooth decay, infection or periodontal reasons? (except wisdom teeth)

Type: Single choice

- none
- from 0 to 2
- from 3 to 5
- from 6 to 10

- more than 10
- all of them

30. Q30. How do oral problems caused by EB impact your life quality? (e.g. not being able to eat what you want, having to give up lunch/dinner with friends, etc.)

Type: 4-point Likert (impact)

- no impact at all
- on a few activities
- on many occasions
- all the time

31. Q31. What are the emotions that you feel when you think about your mouth? (you can select multiple answers)

Type: Multiple choice

- anxiety for the future
- anger
- shame
- guilt
- none

Note: Multiple response; exploratory analyses

Abbreviazioni / Abbreviations
EB = epidermolisi bollosa / epidermolysis bullosa; EBS = EB semplice / simplex; JEB = EB giunzionale / junctional; DDEB = EB distrofica dominante; RDEB = EB distrofica recessiva; KEB = EB di Kindler; PEG = gastrostomia endoscopica percutanea; QoL = qualità della vita; OHRQoL = qualità della vita relativa alla salute orale.
